# A Retrospective Case Series Analysis of the Relationship Between Phenylalanine: Tyrosine Ratio and Cerebral Glucose Metabolism in Classical Phenylketonuria and Hyperphenylalaninemia

**DOI:** 10.3389/fnins.2021.664525

**Published:** 2021-06-17

**Authors:** Colm J. McGinnity, Daniela A. Riaño Barros, Eric Guedj, Nadine Girard, Christopher Symeon, Helen Walker, Sally F. Barrington, Mary Summers, Mervi Pitkanen, Yusof Rahman

**Affiliations:** ^1^King’s College London and Guy’s and St Thomas’ PET Centre, School of Biomedical Engineering and Imaging Sciences, King’s College London, London, United Kingdom; ^2^South London and Maudsley NHS Foundation Trust, London, United Kingdom; ^3^APHM, CNRS, Centrale Marseille, Institut Fresnel, Timone Hospital, CERIMED, Nuclear Medicine Department, Aix-Marseille University, Marseille, France; ^4^Aix-Marseille University, APHM, CNRS, CRMBM, Marseille, France; ^5^Institute of Psychiatry, Psychology & Neuroscience, King’s Health Partners, King’s College London, London, United Kingdom; ^6^West London NHS Trust, London, United Kingdom; ^7^Guy’s and St Thomas’ NHS Foundation Trust, London, United Kingdom

**Keywords:** phenylketonuria, hyperphenylalaninemia, [^18^F]fluorodeoxyglucose PET, phenylalanine:tyrosine ratio, intellectual function

## Abstract

We retrospectively examined the relationship between blood biomarkers, in particular the historical mean phenylalanine to tyrosine (Phe:Tyr) ratio, and cerebral glucose metabolism. We hypothesized that the historical mean Phe:Tyr ratio would be more predictive of cerebral glucose metabolism than the phenylalanine (Phe) level alone. We performed a retrospective case series analysis involving 11 adult classical phenylketonuria/hyperphenylalaninemia patients under the care of an Inherited Metabolic & Neuropsychiatry Clinic who had complained of memory problems, collating casenote data from blood biochemistry, and clinical [^18^F]fluorodeoxyglucose positron emission tomography ([^18^F]FDG PET). The Phe:Tyr ratio was calculated for individual blood samples and summarized as historical mean Phe:Tyr ratio (Phe:Tyr) and historical standard deviation in Phe:Tyr ratio (SD-Phe:Tyr), for each patient. Visual analyses of [^18^F]FDG PET revealed heterogeneous patterns of glucose hypometabolism for eight patients. [^18^F]FDG PET standardized uptake was negatively correlated with Phe in a large cluster with peak localized to right superior parietal gyrus. Even larger clusters of negative correlation that encompassed most of the brain, with frontal peaks, were observed with Phe:Tyr, and SD-Phe:Tyr. Our case series analysis provides further evidence for the association between blood biomarkers, and cerebral glucose hypometabolism. Mean historical blood Phe:Tyr ratio, and its standard deviation over time, appear to be more indicative of global cerebral glucose metabolism in patients with memory problems than Phe.

## Introduction

Phenylketonuria (PKU) is an inherited metabolic disease, with prevalence rates of around 1:10,000 births in Northern Europe (Loeber, 2007). It occurs due to a deficiency in the phenylalanine hydroxylase (PAH) enzyme, which metabolizes the amino acid phenylalanine (Phe) into tyrosine (Tyr). Tyr then undergoes further metabolism into melanin, and thyroxine, and also is a precursor to neurotransmitters including dopamine and adrenaline (Schuck et al., 2015).

Phenylalanine hydroxylase deficiency can occur to varying degrees, resulting in differences in the severity of phenotypes. “Classical” PKU is the most severe of the phenotypes, with a near complete deficiency of PAH activity. Pre-treatment Phe levels over 1200 μmol/L are considered diagnostic for “classical” PKU, whereas levels between 600 and 1200 μmol/L are referred to as “atypical/mild” PKU, and 120–600 μmol/L as (mild) hyperphenylalaninemia (HPA) (Blau et al., 2010). However, in the adult clinical setting, the distinction between HPA and mild PKU phenotypes is often arbitrary due to the lack of historical data, and mostly based on patients’ self-reported natural protein intake/tolerance and their periodic blood Phe.

Following the implementation of national birth screening and dietary control measures, there has been a decrease in the rates of complications resulting from the severe phenotype, which include microcephaly, seizures, behavioral difficulties, reduced intelligence, severe learning disability and tremor (Trefz et al., 2000; Williams et al., 2008). However, the identification of other neurocognitive symptoms has increased. For example, a meta-analysis found a negative correlation between IQ and Phe concentration (Fonnesbeck et al., 2013). Frontal executive dysfunction has also been identified in patients with PKU. Nardecchia et al. (2015) conducted a long term follow up study in which they identified relative impairment of function in executive abilities, visuospatial and attentional skills, compared to controls. The phenylalanine-to-tyrosine (Phe:Tyr) ratio has been correlated with executive function measures, to a greater extent that Phe alone.

Whilst neuroimaging is not routinely performed in phenylketonuria, cerebral glucose utilization has been studied sparingly. In the largest study to date, Wasserstein et al. reported that plasma phenylalanine was negatively correlated with *relative* cerebral glucose metabolic rates, as measured via [^18^F]fluorodeoxyglucose positron emission tomography (PET), in ten patients with classical PKU who performed a verbal learning task during the tracer uptake period. The PKU group also had lower cortical relative glucose metabolic rate than the control group, a finding that was consistent with smaller clinical (Yanai et al., 1987; Hasselbalch et al., 1996; Ficicioglu et al., 2013) and animal studies (Qin and Smith, 2007; Dienel and Cruz, 2016).

It is not yet known whether cerebral glucose metabolism is also reduced in patients with classical PKU or HPA and memory problems. Our case series analysis aimed to investigate the relationships between blood biomarkers, in particular the historical mean Phe:Tyr ratio, and cerebral glucose metabolism. Based on the studies of executive function in phenylketonuria, we hypothesized that the Phe:Tyr ratio would be more predictive of cerebral glucose metabolism than the Phe level alone.

## Materials and Methods

A retrospective case series analysis was performed in 11 adult patients under the care of the Inherited Metabolic & Neuropsychiatry Clinic at St Thomas’ Hospital, London who complained of memory problems. We collated casenote data from neuropsychiatric assessments, blood biochemistry tests, and clinical [^18^F]fluorodeoxyglucose positron emission tomography ([^18^F]FDG PET). Genetic data were unavailable as gene analysis was not routinely performed.

### Ethics Approval and Patient Consent Statement

Ethical approval was not required for this retrospective case series analysis of data that had already been acquired as part of routine clinical care. All patients provided verbal informed consent for the data acquisition as part of routine clinical care. Patients were informed that their anonymized data could be used for research and teaching.

### Blood Biochemistry

Blood biochemistry test results included repeated Phe and Tyr measurements. The Phe:Tyr ratio was calculated for individual blood samples at each time point and subsequently averaged to give a historical mean Phe:Tyr ratio for each patient. We also computed the standard deviation in historical Phe:Tyr ratio for each patient. All blood samples were processed in the same laboratory.

### [^18^F]FDG PET

[^18^F]FDG PET scans were acquired using several different models of the Discovery PET/CT scanner series (ST/STE/710) from GE Healthcare (Little Chalfont, United Kingdom). The images, reconstructed iteratively using ordered subsets expectation maximization (OSEM), were reported clinically by two Consultants in Nuclear Medicine using visual interpretation and re-reviewed at a later stage for the purposes of this case series analysis by SFB to confirm consistency of reporting. In order to allow quantitative analyses, the images initially displayed in absolute counts scaled to the maximum value were displayed as standardized uptake values (SUVs), calculated according to Kenney et al. (1941):

(1) $$S\,U\,V=\frac{R\,a\,d\,i\,o\,a\,c\,t\,i\,v\,i\,t\,y\,c\,o\,n\,c\,e\,n\,t\,r\,a\,t\,i\,o\,n\,\left(K\,B\,q/m\,l\right)*w\,e\,i\,g\,h\,t\,\left(k\,g\right)}{I\,n\,j\,e\,c\,t\,e\,d\,d\,o\,s\,e\,\left(M\,B\,q\right)}$$

The SUV images were subsequently aligned to the [^18^F]FDG PET template image in MNI space provided with SPM12, via non-linear registration. The SUV images were smoothed with an isotropic Gaussian filter of eight millimeters full-width at half-maximum to account for modest differences between scanners.

### Voxel-By-Voxel Analyses

The relationships between SUV and Phe, SUV and the standard deviation in Phe (SD-Phe), SUV and Phe:Tyr, and SUV and the standard deviation in Phe:Tyr (SD-Phe:Tyr), were interrogated separately on a voxel-by-voxel basis via multisubject simple regression using threshold-free cluster enhancement as implemented in FSL version 5.0.9^1^ with the “randomize” tool, version 2.9. We used 10,000 permutations, an explicit whole brain mask, and a stringent threshold of *p* < 0.05 after family-wise error correction.

The patients were also compared on a voxel-by-voxel basis with 25 healthy controls (median ± interquartile range 35.0 ± 12 years, minimum 29 years, maximum 40 years; 13 females) that had been scanned at a different Centre using a GE Discovery ST PET/CT scanner. A two-sample *t*-test was performed using threshold-free cluster enhancement as implemented in FSL-randomize, with an additional age covariate and proportional scaling. The number of permutations, masking and thresholds were described above.

## Results

Six females and five males were included in the case series analysis. Age and blood biochemistry data are summarized in Table 1. Eight patients had a diagnosis of classical PKU and three had a diagnosis of HPA.

**TABLE 1:** Demographics, intellectual functions, and blood biochemistry.

	**Median**	**Interquartile Range**	**Minimum**	**Maximum**
Age at scan	31	13	18	40
Years of education	11	4	10	17
Verbal Comprehension Index	103	13	73	121
Perceptual Reasoning Index	94	15	71	134
Working Memory Index	89	12	74	103
Processing Speed Index	95	16	75	108
Full-scale IQ	92	19	71	107
Phenylalanine at scan (μmol/ml; *n* = 11)	742	434	237	1304
Mean historical phenylalanine (μmol/ml; *n* = 10)	662	314	274	877
Standard deviation in historical phenylalanine (μmol/ml; *n* = 10)	193	95	50	358
Mean historical Phe:Tyr ratio (*n* = 9)	16	11	3	37
Standard deviation in historical Phe:Tyr ratio (*n* = 9)	11	6	1	20

Three patients, one with classical PKU and two with HPA, had stopped adhering to a low Phe diet before reaching 10 years of age; six patients with classical PKU stopped adhering to it between 11 and 20 years of age; and the remaining two patients, one with classical PKU and one with HPA, had adhered to it throughout their life.

Longitudinal historical Phe levels were available for 10 patients (median ± interquartile range 12.5 ± 15 samples, minimum 8, maximum 29) and repeated historical Tyr levels were available for nine (12 ± 15 samples, minimum 6, maximum 25). Therefore, the mean historical Phe:Tyr ratios and standard deviation in Phe:Tyr ratios were calculated for nine patients. Phe levels at the time of scan were available for all 11 patients.

### Neuroimaging Findings

#### Visual Interpretation

**FIGURE 1:**
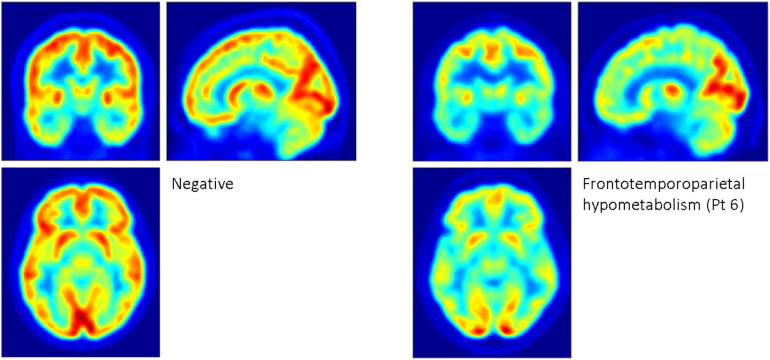
Examples of patterns of glucose metabolism revealed by [^18^F]FDG PET. The panel on the left depicts the mean of two patients who had scans that were reported as normal. The panel on the right depicts marked, widespread frontotemporoparietal hypometabolism sparing the occipital cortex, seen in patient 6, a 40-year-old male with full-scale IQ of 90 who ceased adherence to the low phenylalanine diet before he reached 10 years of age. His most recent blood spot phenylalanine measurement prior to the scan was 1200 μmol/ml. Based on 25 phenylalanine and 22 tyrosine measurements, his mean ± standard deviation historical phenylalanine was 877.2 ± 183.6 μmol/ml, and his Phe:Tyr ratio was 89.2 ± 52.6.

[^18^F]FDG PET revealed heterogeneous patterns of glucose metabolism for eight of the 11 patients (Figure 1). Hypometabolic distributions was summarized as: frontal (2), temporal (1), frontotemporal (1), frontotemporoparietal (3), and temporoparietal (1) patterns. Hypermetabolism was not identified.

#### Voxel-By-Voxel Analyses – Correlations

The SUV was not positively correlated with any of the examined variables.

A significant negative correlation was observed between SUV and Phe, in an extremely large cluster (577,784 mm^3^), with peak localized to the right superior parietal gyrus (Table 2 and Supplementary Material, Figures 1, 2). The cluster encompassed approximately 31% of the brain mask by volume. A second cluster of 1,991 mm^3^ was observed with peak localized to the right caudate nucleus.

**TABLE 2:** Clusters of negative correlation between SUV and Phe, between SUV and Phe:Tyr, and between SUV and SD-Phe:Tyr.

**SUV-Phe**					
		**Peak**		**Centre-of-Gravity**	
	**Volume (mm^3^)**	**x, y, z (mm)**	**Region Name**	**x, y, z (mm)**	**Region Name**
Negative	577,784	42, −38, 42	Right superior parietal gyrus	08, −19, 22	Corpus callosum
	1,992	14, 16, 02	Right caudate nucleus	14, 13, 06	Right caudate nucleus
Positive	–	–	–	–	–

**SUV-Phe:Tyr**					
		**Peak**		**Centre-of-Gravity**	
	**Volume (mm^3^)**	**x, y, z (mm)**	**Region Name**	**x, y, z (mm)**	**Region Name**

Negative	1,603,968	44, 46, −02	Right middle frontal gyrus	03, −23, 10	Third ventricle
Positive	–	–	–	–	–

**SUV-SD-Phe:Tyr**					
		**Peak**		**Centre-of-Gravity**	
	**Volume (mm^3^)**	**x, y, z (mm)**	**Region Name**	**x, y, z (mm)**	**Region Name**

Negative	1,641,632	42, 44, 00	Right middle frontal gyrus	03, −23, 10	Third ventricle
Positive	–	–	–	–	–

There was no significant correlation between SUV and SD-Phe.

A significant negative correlation was observed between SUV and Phe:Tyr, in an extremely large cluster (1,603,968 mm^3^) with peak localized to the right middle frontal gyrus (Table 2 and Supplementary Figures 3, 4). The cluster encompassed approximately 86% of the brain mask.

A significant negative correlation was also observed between SUV and SD-Phe:Tyr, in an extremely large cluster (1,641,632 mm^3^) with peak localized to the right middle frontal gyrus (Table 2 and Supplementary Figures 5, 6). The cluster encompassed approximately 88% of the brain mask.

#### Voxel-By-Voxel Analyses – Comparison With Controls

The patients had significantly lower uptake than controls in five clusters, the largest of which (45,464 mm^3^) had peak in the right inferior and middle temporal gyri (Table 3 and Supplementary Figure 7). The patients had significantly greater uptake than controls in two clusters, the largest of which (135,016 mm^3^) had peak localized to the medial aspect of the right superior frontal gyrus (Table 3 and Supplementary Figure 7).

**TABLE 3:** Clusters of significantly lower and higher [^18^F]FDG uptake in patients, relative to healthy controls.

		**Peak**		**Centre-of-Gravity**	
	**Volume (mm^3^)**	**x, y, z (mm)**	**Region Name**	**x, y, z (mm)**	**Region Name**
**Patients < Controls**	45,464	56, −14, −26	Right inferior and middle temporal gyri	46, −24, −05	Right superior temporal gyrus (middle aspect)
	5,816	−04, −22, −20	Brainstem	01, −24, −29	Brainstem
	3,168	32, 50, 06	Right middle frontal gyrus	29, 47, 03	Right middle frontal gyrus
	672	04, −32, −62	Brainstem	−32, −62, 04	Left posterior temporal lobe
	56	32, 22, 34	Right middle frontal gyrus	33, 21, 35	Right middle frontal gyrus
**Positive**	135,016	04, 40, 26	Right superior frontal gyrus (medial aspect)	−04, −07, 55	Left superior frontal gyrus (medial aspect)
	18,368	06, −88, 10	Right cuneus	03, −73, 05	Right lingual gyrus

## Discussion

The major findings from this retrospective case series analysis of 11 patients were: (1) eight patients had cortical deficits of cerebral glucose metabolism; and (2) [^18^F]FDG SUV (i.e., glucose metabolism) was negatively correlated with Phe, mean historical Phe:Tyr ratio, and standard deviation in historical Phe:Tyr ratio, across much of the brain.

We report *resting* focal hypometabolism on *individual* visual analyses for 8 of 11 patients. In contrast, Wasserstein et al. performed a comparison of relative cerebral glucose metabolic rates (i.e., whole-brain normalized [^18^F]FDG uptake) with healthy controls (Wasserstein et al., 2006), after performance of a verbal learning task during the uptake period. Our individual results also extend the findings of Wasserstein et al. to patients with memory problems, and also to patients with HPA. Whilst we interpret the results of our between-group comparison with caution, given that control data were acquired from a different Centre, we note that like Wasserstein et al. we found bidirectional alterations in uptake.

To our knowledge this is the first finding of a negative correlation between cerebral glucose metabolism and mean Phe:Tyr ratio or standard deviation in Phe:Tyr ratio. Variability in Phe has been found to be a stronger predictor of poor cognitive performance than other indices of Phe control including mean historical Phe (Hood et al., 2014). In our case series analysis the Phe levels measured within three months of the scan did not significantly correlate with cerebral glucose metabolism contrary to Wasserstein et al. (2006; data not shown). However, similar to Wasserstein et al. (2006), we found a significant negative correlation with mean historical Phe. Our findings complement those of Hood et al. (2014) regarding variability and additionally suggest that Phe:Tyr ratio might be more indicative of global cerebral glucose metabolism than Phe. A limitation of the ratio is that blood Tyr is subject to diurnal variation and is also influenced by food intake; however, this influence can be minimized by acquiring samples after fasting and/or averaging several measurements. In those with a high historical Phe:Tyr ratio, we expect that the elevated blood Phe would have decreased the concentration of tyrosine in the brain, due to mutual competition for the large neutral amino acids type I (LAT1) countertransporter at the blood brain barrier. The “dopamine depletion” hypothesis (Diamond et al., 1997) posits that decreased dopamine synthesis results from a state of cerebral tyrosine deficiency and contributes to cognitive dysfunction. Noradrenergic neurotransmission is also expected to be decreased, as it is produced by the metabolism of dopamine. A serotonin deficiency is also expected as tryptophan also competes with Phe for LAT1. Our data additionally suggest that the cerebral tyrosine deficiency is coupled with cerebral glucose hypometabolism, the mechanism of which remains to be elucidated. We speculate that cerebral hypometabolism in classical PKU reflects decreased energy (e.g., dendritic and astrocytic ion pump) usage via dopaminergic (Landvogt et al., 2008; Boot et al., 2017), noradrenergic, and serotonergic neurotransmission. Further exploration of the relationships between blood biomarkers, cerebral glucose hypometabolism, structural [e.g., (Perez-Duenas et al., 2006; Vermathen et al., 2007; Scarabino et al., 2009; Bodner et al., 2012; Christ et al., 2016] and functional magnetic resonance imaging (fMRI; (Christ et al., 2010, 2012, 2013)) abnormalities might help to elucidate the pathophysiological basis of impairment. This might include measurement of concentrations of the dopamine metabolite homovanillic acid and the serotonin metabolite 5-hydroxyindoleacetic acid in cerebrospinal fluid, and of prolactin in serum (secretion of which is inhibited by dopamine).

Our case series analysis is limited due to sample size and the availability of patient data, which we note was hard to avoid given it’s retrospective nature and relative low prevalence of HPA and classical PKU; and by the use of control data from a different Centre. Whilst we did not distinguish between HPA and PKU (for statistical power), and the outcomes of these diseases may differ substantially, all patients complained of memory problems. The retrospective character of our case series analysis constitutes an additional limitation, for example resulting in a lack of data on potential confounds and a lack of standardization during data collection, which precluded meaningful SUV-IQ and SUV-diet correlations. Due to their relatively low prevalence, a prospective multicentre study may be useful to increase power, allow subgroup analyses based on genotype (e.g., of the LAT1 countertransporter) or phenotype, and provide a wider demographic profile, thus increasing validity.

The patients reported in this case series analysis were all attendees of the Inherited Metabolic & Neuropsychiatry Clinic who had complained of memory problems, thus the findings cannot be extrapolated to those without memory complaints. Further studies are required to confirm the findings are applicable to the wider PKU/HPA patient population. It was beyond the scope of our case series analysis to distinguish the Phe:Tyr – cerebral glucose metabolism relationships between co-morbidities of interest, such as affective disorders or executive dysfunction; this could be a valuable insight provided by a multicentre study.

In conclusion, our case series analysis provides further evidence for neurocognitive impairment in patients with classical PKU/HPA, as well as reductions in cerebral glucose hypometabolism. Mean historical blood Phe:Tyr ratio, and its standard deviation, appear to be more indicative of global cerebral glucose metabolism in patients with memory problems than Phe. Our findings emphasize the need for regulation of Phe intake in classical PKU/HPA.

## Data Availability Statement

We are not permitted to make the patient datasets presented in this article available because it was provided as part of routine clinical care – i.e., without consent for sharing widely. Requests for access to the controls dataset should be directed to EG: eric.guedj@univ-amu.fr.

## Ethics Statement

Ethical approval was not required for the retrospective case series analysis of data that had already been acquired as part of routine clinical care. All patients provided verbal informed consent for the data acquisition, and for use of their anonymized data in research and teaching. The controls database was acquired with the approval of approval of “CPP Sud Méditerranée V” ethics committee (clinical trials registration: NCT00484523). All control participants provided informed written consent.

## Author Contributions

CM and DR were responsible for the data analyses and interpretation, and manuscript preparation. EG and NG were responsible for acquisition of data from the healthy controls and contributed to manuscript preparation. CS contributed to the data analyses and interpretation and manuscript preparation. HW contributed to manuscript preparation. SFB contributed to PET analyses and interpretation and manuscript preparation. MS contributed to statistical analyses, explanation and interpretation of the neuropsychological data, and manuscript preparation. MP and YR were responsible for conception and design of the case series analysis. They selected the cases, having previously seen these patients in the Inherited Metabolic & Neuropsychiatry Clinic at GSTT. They also contributed to data acquisition and preparation of the manuscript. All authors contributed to the article and approved the submitted version.

## Conflict of Interest

The authors declare that the research was conducted in the absence of any commercial or financial relationships that could be construed as a potential conflict of interest.

## Footnotes

1 https://fsl.fmrib.ox.ac.uk/fsl/fslwiki

## References

[B1] Blau N. van Spronsen F. J. Levy H. L. (2010). Phenylketonuria. *Lancet* 376 1417–1427. 10.1016/s0140-6736(10)60961-0 20971365

[B2] Bodner K. E. Aldridge K. Moffitt A. J. Peck D. White D. A. Christ S. E. (2012). A volumetric study of basal ganglia structures in individuals with early-treated phenylketonuria. *Mol. Genet. Metab.* 107 302–307. 10.1016/j.ymgme.2012.08.007 23006929

[B3] Boot E. Hollak C. E. M. Huijbregts S. C. J. Jahja R. van Vliet D. Nederveen A. J. (2017). Cerebral dopamine deficiency, plasma monoamine alterations and neurocognitive deficits in adults with phenylketonuria. *Psychol. Med.* 47, 2854–2865. 10.1017/S0033291717001398 28552082

[B4] Christ S. E. Moffitt A. J. Peck D. (2010). Disruption of prefrontal function and connectivity in individuals with phenylketonuria. *Mol. Genet. Metab.* 99(Suppl. 1) S33–S40. 10.1016/j.ymgme.2009.09.014 20123468

[B5] Christ S. E. Moffitt A. J. Peck D. White D. A. (2013). The effects of tetrahydrobiopterin (BH4) treatment on brain function in individuals with phenylketonuria. *Neuroimage Clin.* 3 539–547. 10.1016/j.nicl.2013.08.012 24371792 PMC3871382

[B6] Christ S. E. Moffitt A. J. Peck D. White D. A. Hilgard J. (2012). Decreased functional brain connectivity in individuals with early-treated phenylketonuria: evidence from resting state fMRI. *J. Inherit. Metab. Dis.* 35 807–816. 10.1007/s10545-011-9439-9 22231384

[B7] Christ S. E. Price M. H. Bodner K. E. Saville C. Moffitt A. J. Peck D. (2016). Morphometric analysis of gray matter integrity in individuals with early-treated phenylketonuria. *Mol. Genet. Metab.* 118 3–8. 10.1016/j.ymgme.2016.02.004 26947918

[B8] Diamond A. Prevor M. B. Callender G. Druin D. P. (1997). Prefrontal cortex cognitive deficits in children treated early and continuously for PKU. *Monogr. Soc. Res. Child. Dev.* 62 i–v, 1–208. 10.2307/1166208 9421921

[B9] Dienel G. A. Cruz N. F. (2016). Biochemical, metabolic, and behavioral characteristics of immature chronic hyperphenylalanemic rats. *Neurochem. Res.* 41 16–32. 10.1007/s11064-015-1678-y 26224289 PMC4733436

[B10] Ficicioglu C. Dubroff J. G. Thomas N. Gallagher P. R. Burfield J. Hussa C. (2013). A pilot study of fluorodeoxyglucose positron emission tomography findings in patients with phenylketonuria before and during sapropterin supplementation. *J. Clin. Neurol.* 9 151–156. 10.3988/jcn.2013.9.3.151 23894238 PMC3722466

[B11] Fonnesbeck C. J. McPheeters M. L. Krishnaswami S. Lindegren M. L. Reimschisel T. (2013). Estimating the probability of IQ impairment from blood phenylalanine for phenylketonuria patients: a hierarchical meta-analysis. *J. Inherit. Metab. Dis.* 36 757–766. 10.1007/s10545-012-9564-0 23197105

[B12] Hasselbalch S. Knudsen G. M. Toft P. B. Høgh P. Tedeschi E. Holm S. (1996). Cerebral glucose metabolism is decreased in white matter changes in patients with phenylketonuria. *Pediatr. Res.* 40 21–24. 10.1203/00006450-199607000-00004 8798240

[B13] Hood A. Grange D. K. Christ S. E. Steiner R. White D. A. (2014). Variability in phenylalanine control predicts IQ and executive abilities in children with phenylketonuria. *Mol. Genet. Metab.* 111 445–451. 10.1016/j.ymgme.2014.01.012 24568837 PMC4144445

[B14] Kenney J. M. Marinelli L. Woodard H. Q. (1941). Tracer studies with radioactive phosphorus in malignant neoplastic disease. *Radiology* 37 683–690. 10.1148/37.6.683

[B15] Landvogt C. Mengel E. Bartenstein P. Buchholz H. G. Schreckenberger M. Siessmeier T. (2008). Reduced cerebral fluoro-L-dopamine uptake in adult patients suffering from phenylketonuria. *J. Cereb Blood Flow Metab.* 28, 824–831. 10.1038/sj.jcbfm.9600571 17971791

[B16] Loeber J. G. (2007). Neonatal screening in Europe; the situation in 2004. *J. Inherit. Metab. Dis.* 30 430–438. 10.1007/s10545-007-0644-5 17616847

[B17] Nardecchia F. Manti F. Chiarotti F. Carducci C. Carducci C. Leuzzi V. (2015). Neurocognitive and neuroimaging outcome of early treated young adult PKU patients: a longitudinal study. *Mol. Genet. Metab.* 115 84–90. 10.1016/j.ymgme.2015.04.003 25952249

[B18] Perez-Duenas B. Pujol J. Soriano-Mas C. Ortiz H. Artuch R. Vilaseca M. A. (2006). Global and regional volume changes in the brains of patients with phenylketonuria. *Neurology* 66 1074–1078. 10.1212/01.wnl.0000204415.39853.4a 16606920

[B19] Qin M. Smith C. B. (2007). Regionally selective decreases in cerebral glucose metabolism in a mouse model of phenylketonuria. *J. Inherit. Metab. Dis.* 30 318–325. 10.1007/s10545-007-0583-1 17457692

[B20] Scarabino T. Popolizio T. Tosetti M. Montanaro D. Giannatempo G. M. Terlizzi R. (2009). Phenylketonuria: white-matter changes assessed by 3.0-T magnetic resonance (MR) imaging, MR spectroscopy and MR diffusion. *Radiol. Med.* 114 461–474. 10.1007/s11547-009-0365-y 19277839

[B21] Schuck P. F. Malgarin F. Cararo J. H. Cardoso F. Streck E. L. Ferreira G. C. (2015). Phenylketonuria pathophysiology: on the role of metabolic alterations. *Aging. Dis.* 6 390–399. 10.14336/ad.2015.0827 26425393 PMC4567221

[B22] Trefz F. K. Cipcic-Schmidt S. Koch R. (2000). Final intelligence in late treated patients with phenylketonuria. *Eur. J. Pediatr.* 159(Suppl. 2) S145–S148. 10.1007/pl00014379 11043161

[B23] Vermathen P. Robert-Tissot L. Pietz J. Lutz T. Boesch C. Kreis R. (2007). Characterization of white matter alterations in phenylketonuria by magnetic resonance relaxometry and diffusion tensor imaging. *Magn. Reson. Med.* 58 1145–1156. 10.1002/mrm.21422 18046700

[B24] Wasserstein M. P. Snyderman S. E. Sansaricq C. Buchsbaum M. S. (2006). Cerebral glucose metabolism in adults with early treated classic phenylketonuria. *Mol. Genet. Metab.* 87 272–277. 10.1016/j.ymgme.2005.06.010 16343970

[B25] Williams R. A. Mamotte C. D. Burnett J. R. (2008). Phenylketonuria: an inborn error of phenylalanine metabolism. *Clin. Biochem. Rev.* 29 31–41. 18566668 PMC2423317

[B26] Yanai K. Iinuma K. Matsuzawa T. Ito M. Miyabayashi S. Narisawa K. (1987). Cerebral glucose utilization in pediatric neurological disorders determined by positron emission tomography. *Eur. J. Nucl. Med.* 13 292–296. 10.1007/bf00256553 3499326

