# Supplementary material for: A Retrospective Case Series Analysis of the Relationship Between Phenylalanine: Tyrosine Ratio and Cerebral Glucose Metabolism in Classical Phenylketonuria and Hyperphenylalaninemia
Source: Front Neurosci. 2021 Jun 17;15:664525. doi: 10.3389/fnins.2021.664525 (PMC8248344; doi:10.3389/fnins.2021.664525)
Supplement: Supplementary file 1 [file Data_Sheet_1.PDF]

Supplementary

1 Supplementary Figures

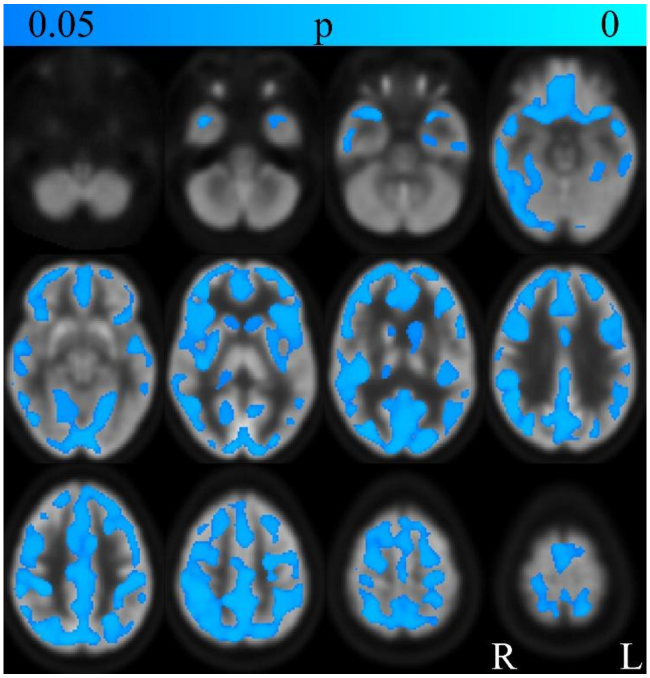

Supplementary Figure 1. Clusters of negative correlation between SUV and Phe, displayed on the SUV template image. L – left; R – right.

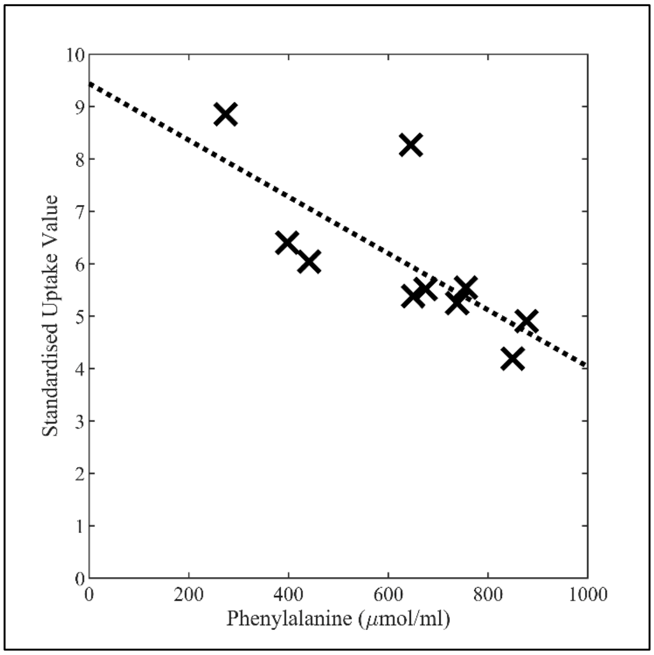

Supplementary Figure 2. Negative correlation between SUV and Phe. The SUVs depicted in the graph represent the median within a sphere of 8 millimeter radius centered on the peak voxel (Spearman's rank ρ = -0.87, p = 0.003).

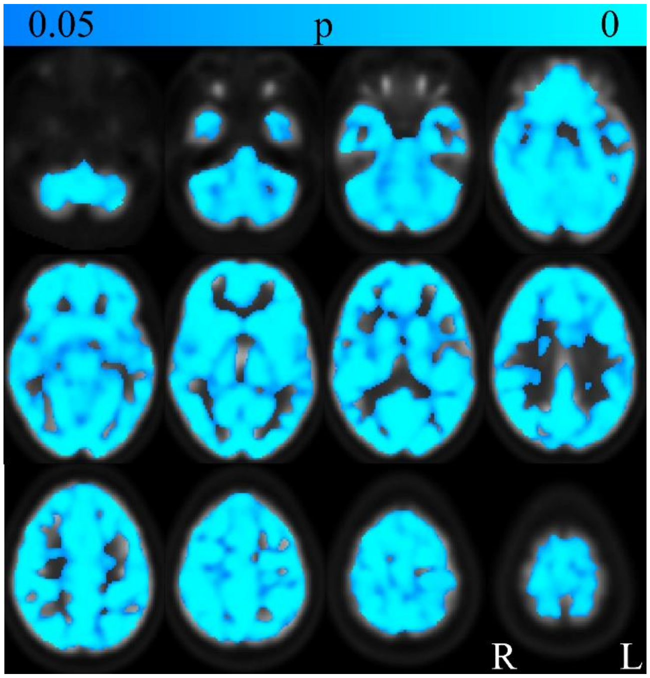

Supplementary Figure 3. Clusters of negative correlation between SUV and Phe:Tyr, displayed on the SUV template image. L – left; R – right.

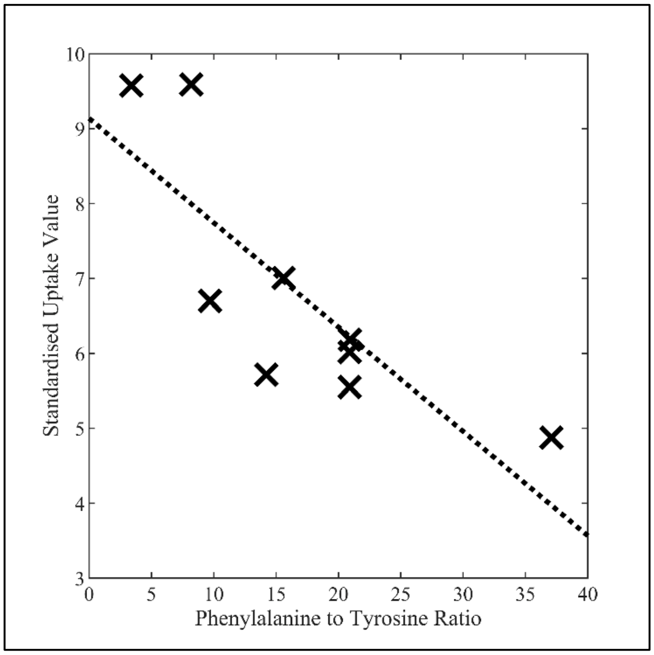

Supplementary Figure 4. Negative correlation between SUV and Phe:Tyr. The SUVs depicted in the graph represent the median within a sphere of 8 millimeter radius centered on the peak voxel (Spearman's rank ρ = -0.75, p = 0.026).

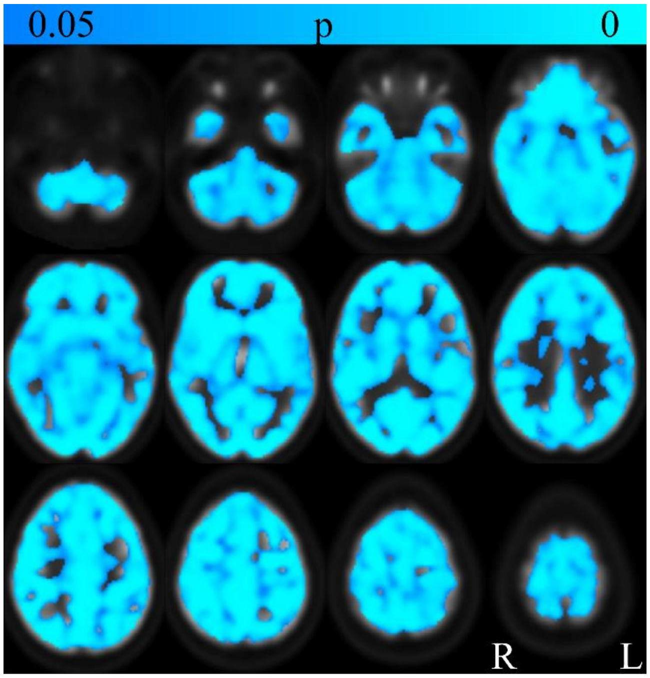

Supplementary Figure 5. Clusters of negative correlation between SUV and SD-Phe:Tyr, displayed on the SUV template image. L – left; R – right.

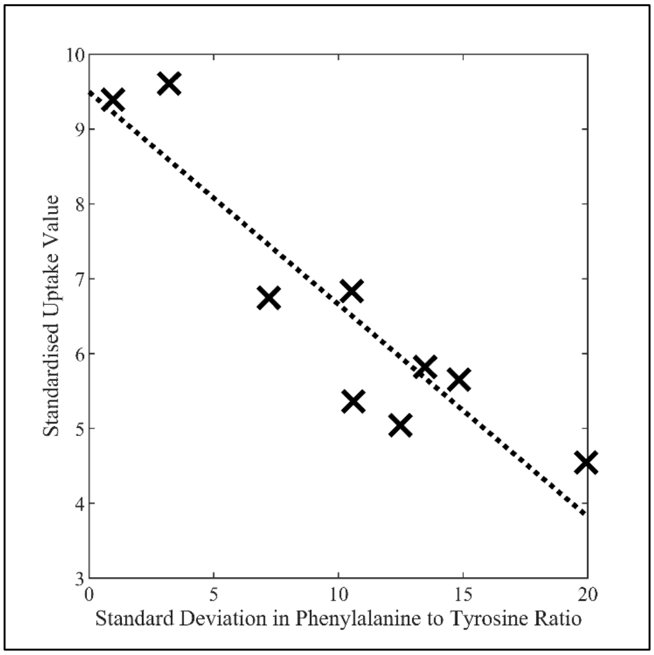

Supplementary Figure 6. Negative correlation between SUV and SD-Phe:Tyr. The SUVs depicted in the graph represent the median within a sphere of 8 millimeter radius centered on the peak voxel (Spearman's rank ρ = -0.83, p = 0.008).

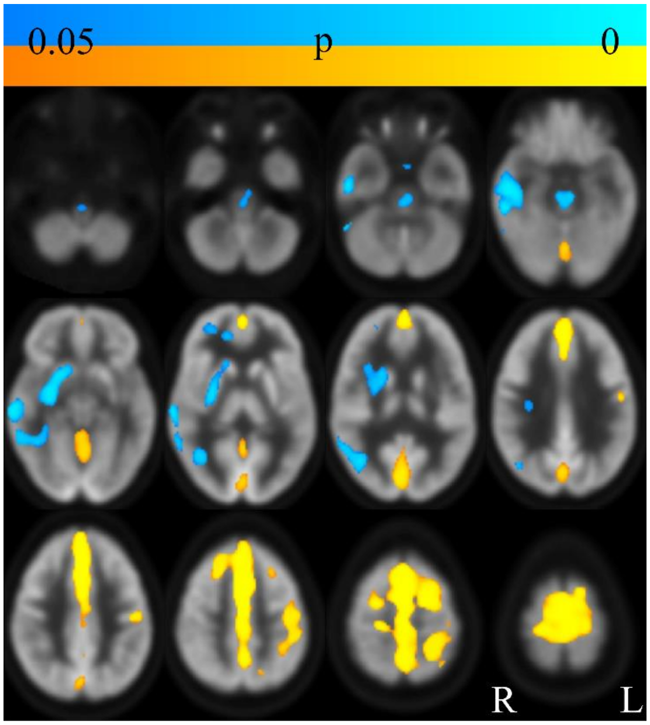

Supplementary Figure 7. Clusters of significantly lower (blue) and higher (yellow) [^{18}F]FDG uptake, relative to healthy controls. L – left; R – right.
